# Exceptional error minimization in putative primordial genetic codes

**DOI:** 10.1186/1745-6150-4-44

**Published:** 2009-11-19

**Authors:** Artem S Novozhilov, Eugene V Koonin

**Affiliations:** 1 National Center for Biotechnology Information, National Library of Medicine, National Institutes of Health, Bethesda, MD 20894, USA

## Abstract

**Background:**

The standard genetic code is redundant and has a highly non-random structure. Codons for the same amino acids typically differ only by the nucleotide in the third position, whereas similar amino acids are encoded, mostly, by codon series that differ by a single base substitution in the third or the first position. As a result, the code is highly albeit not optimally robust to errors of translation, a property that has been interpreted either as a product of selection directed at the minimization of errors or as a non-adaptive by-product of evolution of the code driven by other forces.

**Results:**

We investigated the error-minimization properties of putative primordial codes that consisted of 16 supercodons, with the third base being completely redundant, using a previously derived cost function and the error minimization percentage as the measure of a code's robustness to mistranslation. It is shown that, when the 16-supercodon table is populated with 10 putative primordial amino acids, inferred from the results of abiotic synthesis experiments and other evidence independent of the code's evolution, and with minimal assumptions used to assign the remaining supercodons, the resulting 2-letter codes are nearly optimal in terms of the error minimization level.

**Conclusion:**

The results of the computational experiments with putative primordial genetic codes that contained only two meaningful letters in all codons and encoded 10 to 16 amino acids indicate that such codes are likely to have been nearly optimal with respect to the minimization of translation errors. This near-optimality could be the outcome of extensive early selection during the co-evolution of the code with the primordial, error-prone translation system, or a result of a unique, accidental event. Under this hypothesis, the subsequent expansion of the code resulted in a decrease of the error minimization level that became sustainable owing to the evolution of a high-fidelity translation system.

**Reviewers:**

This article was reviewed by Paul Higgs (nominated by Arcady Mushegian), Rob Knight, and Sandor Pongor. For the complete reports, go to the Reviewers' Reports section.

## Background

The standard genetic code, which is a mapping of 64 codons to 20 standard amino acids and the translation stop signal, is shared, with minor modifications only, by all life forms on earth [1-4]. The apparent universality of the code implies that the last universal common ancestor (LUCA) of all extant life forms should have already possessed, together with a complex translation machinery, the same genetic code as contemporary organisms. One of the central principles of Darwinian evolution is that complex systems evolve from simple ancestors, typically if not always, via a succession of relatively small, incremental steps each of which increases fitness or at least does not lead to a decrease in fitness [5]. In conformity with this continuity principle [6,7], it appears almost certain that the genetic code employed by the primordial translation system was substantially simpler than the modern code, which then evolved incrementally. The origin and evolution, if any, of the genetic code represent a major puzzle of modern biology; numerous hypotheses have been formulated but to date no generally accepted consensus has been reached [8-13].

Several lines of evidence have been used to classify the standard 20 amino acids into 'early' and 'late' ones. The most straightforward indications, conceivably, come from experiments on abiogenic synthesis of organic molecules under supposedly realistic prehistoric atmosphere conditions and external energy sources, a research direction pioneered by Miller and Urey in the 1950s [14-16]. The experiments of Miller and similar experiments subsequently performed by other groups under various models of the ancient atmosphere and using different energy sources, such as spark discharges, ultraviolet light, or irradiation with high energy charged particles [17-19] yielded up to 10 standard amino acids (reviewed in [20]). In general, the results of these experiments are remarkably coherent and lead to the same list of standard amino acids that can be produced under emulated primordial conditions:(1)

The second line of evidence is more speculative in nature and is based on the notion of the precursor-product pairs of amino acids. According to the coevolution theory of the genetic code, the present day amino acids that are used in translation are divided into two phases: phase 1 amino acids came from prebiotic synthesis, and phase 2 amino acids are entirely biogenic and were recruited into the code after the evolution of the respective biosynthetic pathways [10,21]. Strikingly, the list of phase 1 amino acids that was derived from the analysis of biosynthetic pathways completely coincides with the above set of 10 amino acids observed in prebiotic amino acid formation experiments [22]. Furthermore, these 10 amino acids have the lowest free energies of formation, an observation that is compatible with abiogenic emergence [20,23].

Many attempts have been made to derive a universal order of the recruitment of amino acids during evolution [24,25]. Using a combination of 60 different criteria, Trifonov reconstructed a 'consensus temporal order of amino acids' [25]. Although this consensus order has been criticized on several grounds [26], it should be noted that the resulting list of amino acids is in a nearly perfect agreement with the combined results of Miller and Urey type experiments. All amino acids synthesized under putative primordial conditions are classified as 'early' in the consensus analysis, with one minor change: **Ile **is considered to be a 'late' amino acid whose appearance is predated by **Arg **and **Asn **(see below).

On the strength of the consensus order, the results of Miller-type experiments, free energies of formation, and the precursor-product relationship between amino acids, it seems most likely that, although we generally cannot give an exact order of appearance of amino acids in the genetic code, that the primordial genetic code should have coded for a subset of the present day amino acid repertoire, and this subset, probably, included the 10 amino acids in list (1).

The genetic code is a mapping of the set of 64 codons onto the set of 20 standard amino acids used in protein translation (and the stop signal). The continuity principle along with the classification of amino acids into early and late ones suggests that the primordial genetic code specified fewer amino acids than the universal standard code which immediately implies that the ancestral code was even more degenerate than the modern one. Importantly, there is essentially no doubt that, from the very emergence of the code, mRNAs (or, possibly, even chemically different primordial templates) were translated by triplets of nucleotides, even if only a few amino acids were encoded. Any speculation on a primordial code with singlet or doublet codons faces the apparently insurmountable obstacle of the subsequent code expansion to the present day triplet form, which obviously would be effectively fatal [27]. Furthermore, the three-base codon structure of the genetic code is likely to be determined by the physics of the interaction between monomers [28,29] and/or by possibility of simultaneous binding of two RNA adaptors on mRNA [27,30]. If the code always consisted of triplets but specified 16 or fewer amino acids, it appears likely that only the first two bases of each codon were informative in the primordial code whereas the third base did not contribute to coding. In other word, the primordial mRNA sequences would have the form XYNXYNXYN... where X, Y are 'meaningful' nucleotides, and N stands for any nucleotide [30-33]. That the primordial code would have this particular organization is strongly suggested by the structure of the extant code in which redundancy is concentrated almost entirely in the third base; apparently, it is the first and, especially, the second bases that ensure the stability of the interaction between codons and cognate anticodons [30].

It is therefore not unrealistic to propose that the primordial genetic code consisted of 16 supercodons (4-codon series, XYN) and encoded 16 or fewer amino acids, possibly, the 10 inferred early amino acids listed above (1). Here we investigate the properties of such putative primordial codes and show that, under some additional, simple assumptions, they would possess extraordinary error minimization properties.

## Results and Discussion

Assuming that, at a particular early stage of evolution, the primordial genetic code consisted of 16 supercodons, we postulate the following 'parsimony principle':

*If the primordial code encoded an amino acid, then this amino acid was encoded by the same supercodon (four-codon series) that encodes the same amino acid in the standard genetic code (or, at least, a subset of the series encodes the same amino acid)*.

The expansion of the code from codons with two meaningful letters to codons with three meaningful letters is required to involve the minimum possible number of amino acid reassignments; accordingly, expansion of the code only allows recruitment of a subset of codons in a supercodon for a new amino acid but not reassignment of codons within the primordial set of amino acids. This assumption is natural because reassignment of amino acids between supercodons series, obviously, is substantially more disruptive than capturing new amino acids within pre-existing codon series [13]. With one exception, there are no contradictions between the list of putative ancestral amino acids (1) and the parsimony principle: most of the 'early' amino acids are encoded by four-codon series, and only two, **Asp **and **Glu**, do not satisfy the two-letter code scheme and the parsimony principle in that they are encoded by the same supercodon. Following the suggestion of Travers [30], we speculate that decoding of the supercodon GAN initially was stochastic, that is, these very similar amino acids were incorporated more or less randomly in response to the codons of this series, and differentiation of **Asp **and **Glu **was established only after the expansion of the genetic code to three-letter codons.

**Figure 1:**
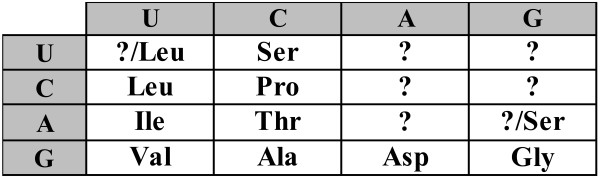
**A 2-letter code consisting of 16 supercodons with the assignment inferred from the list of 'early' amino acids (1) and the parsimony principle**.

Using the parsimony principle, the primordial two-letter code can be partially reconstructed as shown in Fig. 1. Obviously, the parsimony principle does not allow one to infer the assignment for those supercodons that, in the standard code, do not encode any of the primordial amino acids (question marks in Fig. 1). To fill these gaps, additional assumptions on the amino acid assignments are required.

**Figure 2:**
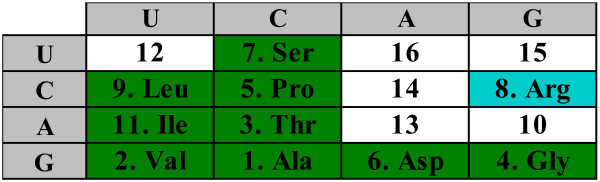
**The order of stabilities of base interaction in the first two codon positions of the standard code according to Travers **[30]. The green highlighting shows the cells that correspond to the supercodons encoding the 'early' amino acids (1). The only difference from the list (1) is shown in blue.

It is instructive to compare the putative core of the primordial genetic code in Fig. 1 with the order of stabilities of the interactions between the first two bases of codons and the cognate anticodons [30] (Fig. 2). There is a striking congruence between the two lists of amino acids. Indeed, the supercodons for 10 early amino acids include 9 of the top 10 most strongly interacting dinucleotides as determined by the stacking and melting thermostabilities. The sole exception is the supercodon CGN that encodes **Arg**, not an early amino acid, but is more stable than CUN and AUN which encode the early amino acids **Leu **and **Ile**, respectively (Fig. 2).

The standard genetic code is manifestly non-random. In particular, the assignments of amino acids to codons are such that the detrimental effect of mistranslation and/or mutation is minimized. That is, in the standard genetic code, codons that differ by one nucleotide code for physicochemically similar amino acids, thus reducing the cost of possible mistranslations and mutations. Quantitative evidence in support of this error-minimization property comes from the comparison of the standard code with random alternatives [11,34-36]. It is thus necessary, when considering any scenario for the origin and evolution of the code, to account for this property. There are two possible explanations for error minimization in the code. The first possibility is that the high degree of error minimization is a byproduct of other processes that shaped the structure of the genetic code (e.g., [13,37,38]). The alternative is the error-minimization (adaptive) theory of the code's evolution which posits that the code evolved under the selective pressure to reduce the consequences of mistranslations and/or mutations [39]. Here we use the same quantitative approach ([11] and see Methods for details) to estimate the error-minimization level of the putative primordial 'two-letter' codes that have at their core the amino acid assignments shown in Fig. 1.

**Figure 3:**
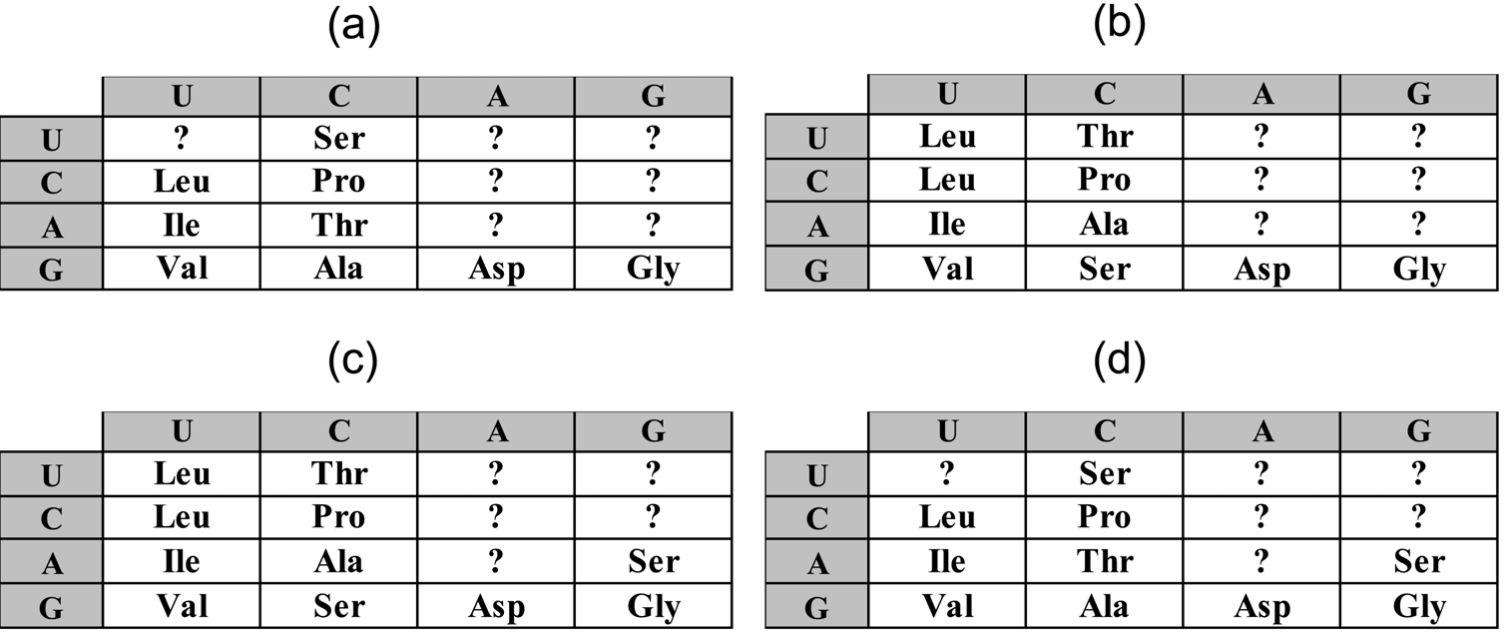
**Error minimization level of 2-letter codes**. Starting from a random permutation of amino acids assignments within the supercodons without question marks, optimization was performed to find the least costly assignment according (see Methods). (a) 9 amino acids, the optimum coincides with the code in Fig. 1, MP = 1; (b) additional assignment of **Leu **is included; the optimum differs from the code in Fig. 1 by permutations in the second column, MP = 0.986; (c) **Leu **and **Ser **are added, MP = 0.985; (d) only **Ser **is added, the optimum is the same code as in Fig. 1, MP = 1.

For the time being, let us disregard the unassigned entries in the code table (question marks in Fig. 1). For any permutation of the amino acid assignments in the code table, a code cost can be calculated. This cost depends on the probability of a given mistranslation error and on the relative cost associated with the replacement of the corresponding wild-type amino acid with a new one (see Methods for the exact details of the calculation of the code cost). Disregarding the unassigned supercodons but otherwise allowing all permutations of amino acid assignments within the rest of the supercodons (9, 10 or 11, depending on whether amino acids are assigned to the UUN and AGN supercodons or not), we find that the code structure in Fig. 1 is close to optimal in terms of error minimization. More precisely, the code structure in Fig. 1 is extremely robust to translational errors irrespective of the assignments of the UUN or AGN supercodons. In two of the four possible cases (Fig. 3a and 3d), there is no permutation that would reduce the cost of the code, that is, the minimization percentage (MP; see Methods for details) of the code is 1; in the other two cases, the optimal codes differ from the code in Fig. 1 only by permutations in the second column, and the MP of these codes is greater than 0.98 (Fig. 3b and 3c).

**Figure 4:**
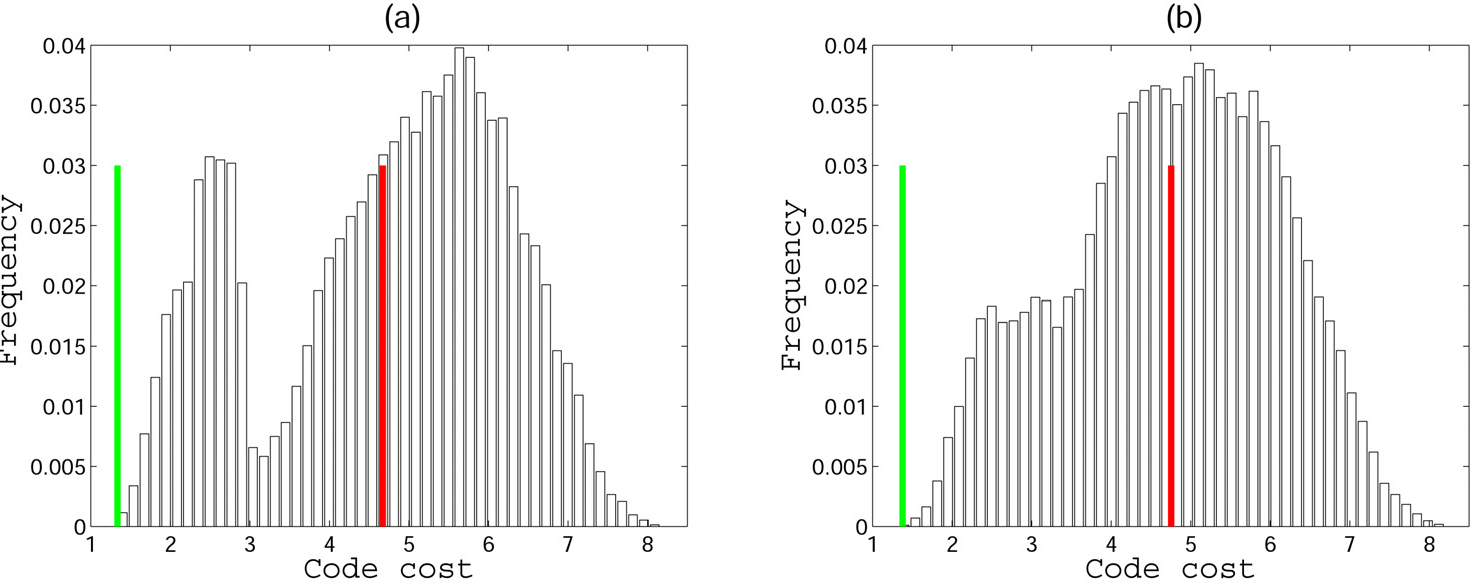
**Distributions of random code costs obtained by permutation of amino acid assignments to the supercodons without question marks in Fig. 2**. The green line shows the cost of the code from Fig. 1, and the red line shows the mean of the distribution. (a) 9 amino acids from Fig. 2a are used, the distance from the mean to the red line is 2.2 standard deviations; (b) **Ser **is added to the list of the 9 amino acids, the distance from the mean to the red line is 2.5 standard deviations.

One possible interpretation of the high robustness of the doublet codes shown in Fig. 3 could be that, with this particular choice of amino acids and supercodons, and the employed measure of the code cost, most of the random codes yield low cost. However, this is not the case, as can be seen from the distribution of random code costs shown in Fig. 4, for the versions of the code from Figs. 3a and 3d. Interestingly, the cost distribution for the code from Fig. 3a is bimodal (a similar distribution was obtained for the code in Fig. 3b; not shown) whereas the distribution for the code from Fig. 3d is a more typical, roughly bell-shaped one. The difference between the cost of the standard code (Fig. 1) and the means of the distributions measured in standard deviations is 2.2, 2.65, 2.91, and 2.5 for the cases (a), (b), (c), (d) in Fig. 3, respectively. Even in the cases (b) and (c), where the assignment of amino acids to supercodons could be improved, the code structure in Fig. 1 is extremely close to the optimum (that, the global cost minimum).

Thus, we showed that the part of the putative two letter primordial genetic code that can be unambiguously inferred assuming the list of early amino acids (1) and the parsimony principle is, in effect, optimal with respect to error minimization property. It seem virtually impossible to explain away this 'perfect' structure as a by-product of some evolutionary process for which error minimization is of secondary importance or neutral. Neither is it possible to explain these codon assignments by random effects because, for instance, for the code in Fig. 3a, there are 181440 (9!/2) alternatives all of which are worse than the one shown in the figure.

**Figure 5:**
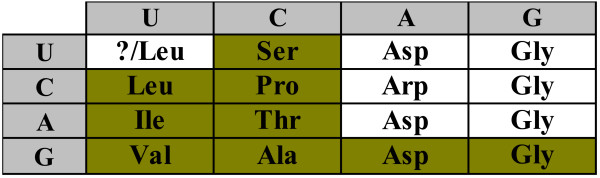
**A 2-letter code consisting of 16 supercodons assigned according to the list of the 'early' amino acids (1), the parsimony principle, and the '4-column' theory**. Dark green cells are those that are assigned in Fig. 1, and question marks in Fig. 1 are replaced with amino acid assignments from the respective column.

**Figure 6:**
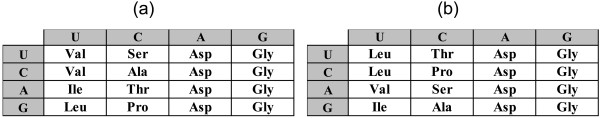
**Error minimization level of 2-letter codes**. Starting from a random permutation of amino acids assignments among the supercodons highlighted in Fig. 5, optimization was performed to find the least costly assignment. (a) 9 amino acids, the optimum code is shown, MP = 0.94; (b) The number of supercodons coding for **Leu **is fixed to 2; MP = 0.987.

**Figure 7:**
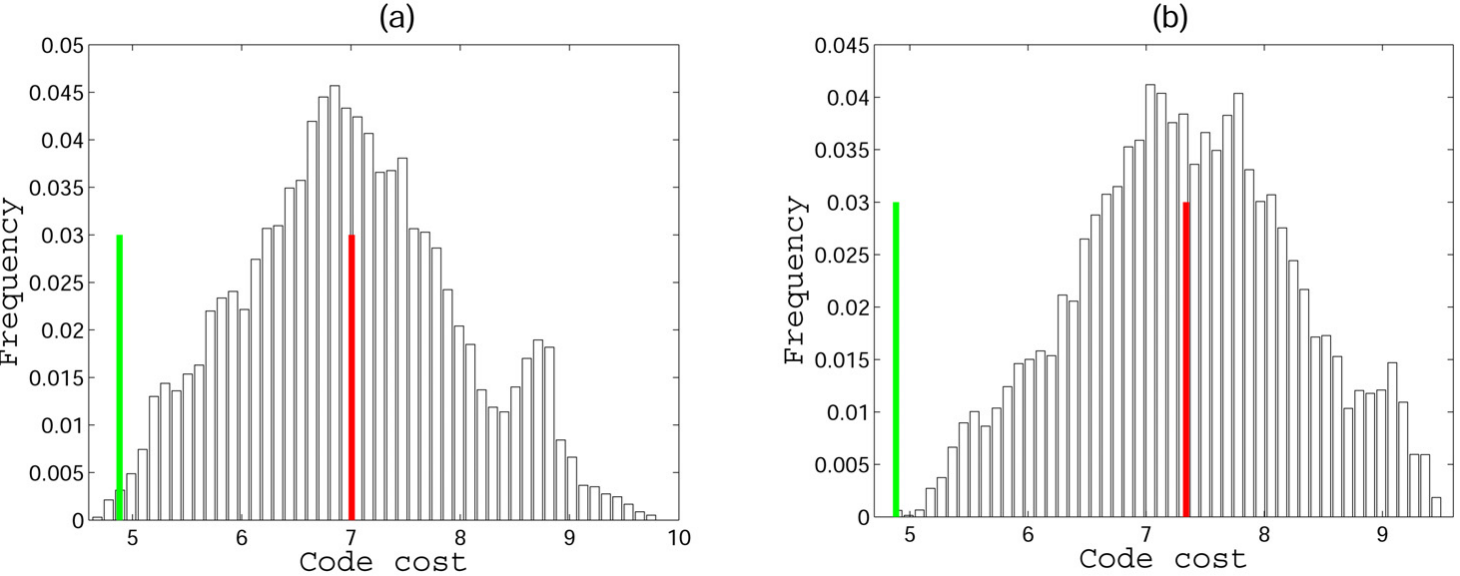
**The distributions of random code costs for the experiments in Fig. 6**. The green line shows the cost of the code from Fig. 5, the red line shows the mean of the distributions. (a) 9 amino acids from Fig. 5, the distance from the mean to the red line is 2.16 standard deviations; (b) Two supercodons assigned for **Leu**; the distance from the green line to the mean is 2.6 standard deviations.

There is, of course, a major caveat in these conclusions. The code cost function is not linear in the sense that adding another amino acid generally destroys the optimal assignments. Given that we disregarded some of the supercodons when performing the numerical experiments described above, the observed extreme error minimization of the putative primordial 2-letter code might be illusory. Therefore, additional assumptions were necessary to fill those supercodons of the 2-letter codes which do not have amino acid assignments after applying of the parsimony principle to the standard code given the list of early amino acids (1). A possible solution that we consider first, is to fill unassigned cells with the amino acids from the same column, in accordance with the 'four-column' theory of the origin of the genetic code [13,40]. For instance, consider the code in Fig. 5. We take the amino acid assignments from Fig. 1 whenever possible, disregard **Ser **for supercodon AGN, so that the whole column codes for the same amino acid, and either assign **Leu **to UUN, because the closest amino acid in this code is **Leu**, or assume the existence of two supercodons for **Leu **(incidentally, the most abundant amino acid in extant proteins) already at the 2-letter stage of the code's evolution. Allowing random permutations of amino acid assignments within the colored cells in Fig. 5 and filling other cells using the 'column-wise' approach, the error minimization properties of the code in Fig. 5 can be assessed. It turns out that the code in Fig. 5 is also highly robust although not quite at the level of the abridged codes in Fig. 3 (Fig. 6). Specifically, if supercodon UUN is filled using the assignment of CUN (**Val**), the MP of the code from Fig. 5 is 0.94 (Fig. 6a); if two supercodons for **Leu **are assumed, then the MP is 0.987, and the optimal code is very close to that in Fig. 5 (Fig. 6b). In both cases, lowest cost was obtained for the assignments where the third and fourth columns code for **Asp **and **Gly**, respectively. The distributions of the random code costs are shown in Fig. 7.

Thus, at least, the part of the 2-letter code that can be inferred from the standard code using the set of (putative) primordial amino acids, the parsimony principle, and a straightforward additional assumption for the assigning the remaining supercodons, is structured in such a way that an *a priori *chosen standard cost function (see Methods) renders the code near-optimal. Indeed, the most conservative estimates yield MP > 0.98 for the cases when the question marks Fig. 1 are disregarded, and MP > 0.94 when the 'four-column' theory is used to assign amino to the unassigned supercodons (Fig. 6), in a sharp contrast to the 78% MP for the standard code [12] (this estimate was obtained using the same cost function as described in the Methods section but for the complete, standard genetic code, and is somewhat higher than the previously reported estimates [41]).

**Figure 8:**
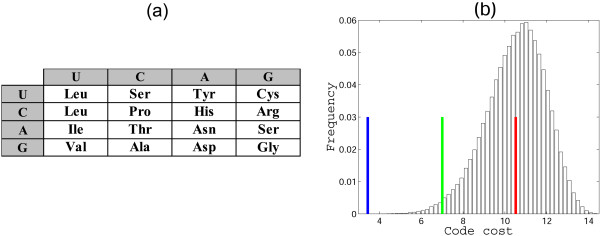
**Error minimization level of 2-letter codes**. (a) A 2-letter code obtained using the parsimony principle. For the cells with an ambiguous assignment, one random amino acid is chosen; (b) The distribution of the costs of the random 2-letter codes obtained by permutation of amino acid assignments in (a), the green lines shows the cost of the code from (a), the red line shows the mean, and the blue line shows the cost of the optimum; MP = 0.51, the distance from the mean is 2.6 standard deviations.

A different approach to assigning the vacant supercodons in the 2-letter in Fig. 1 involves using the parsimony principle not only for the putative early amino acids but for all supercodons. Under this strategy the 2-letter codes cease being special with respect to error-minimization. Consider, for instance, the code shown in Fig. 8a that obtained from the standard code using the parsimony principle. This version of the 2-letter code was proposed as a possible ancestral code [42] and was analyzed with respect to error minimization [43]. This code has MP of 0.51, and the result does not change qualitatively when ambiguous amino acid assignments are changed (for instance, when **Gln **is substituted for **His**). Here our conclusion is in agreement with the conclusions of Butler et al. [43] that were obtained using a different cost function.

With regard to the low error minimization in 2-letter codes obtained using the parsimony principle, we were interested in determining which amino acid assignments contributed the most to this non-optimality. In the standard genetic code, the most non-optimally assigned amino acid is **Arg **[11]; the underlying reason is not only the placement of **Arg **in the code table as such but also the fact that Arg has 6 codons and so makes a disproportionate contribution to the cost of the code. In 2-letter codes, an amino acid can be encoded by two supercodons at the most, so it would not be surprising if an amino acid(s) other than **Arg **occupied the 'worst' position from the point of view of the error minimization.

To address this question for 2-letter codes but taking into account all 20 standard amino acids, we devised the following experiment: for a given natural number *N *≤ 16, choose randomly *N *cells in the 16-cell code table. Then assign amino acids to the chosen cells according to the parsimony principle (if for some cells two amino acids are encoded in the respective 4-codon series in the standard code, one is randomly chosen). Allowing permutations of amino acid assignments between these fixed *N *cells, we can estimate the MP for a given code. Other cells, not chosen in the experiment, can be disregarded, as it was done for the code in Fig. 1, or filled by using, e.g., the four-column rule specified above, as in Fig. 5. Repeating this procedure and collecting random codes with high MP, we can rank the amino acids by the frequency with which they are found in highly optimized codes and similarly rank the cells (supercodons) in the code table.

**Figure 9:**
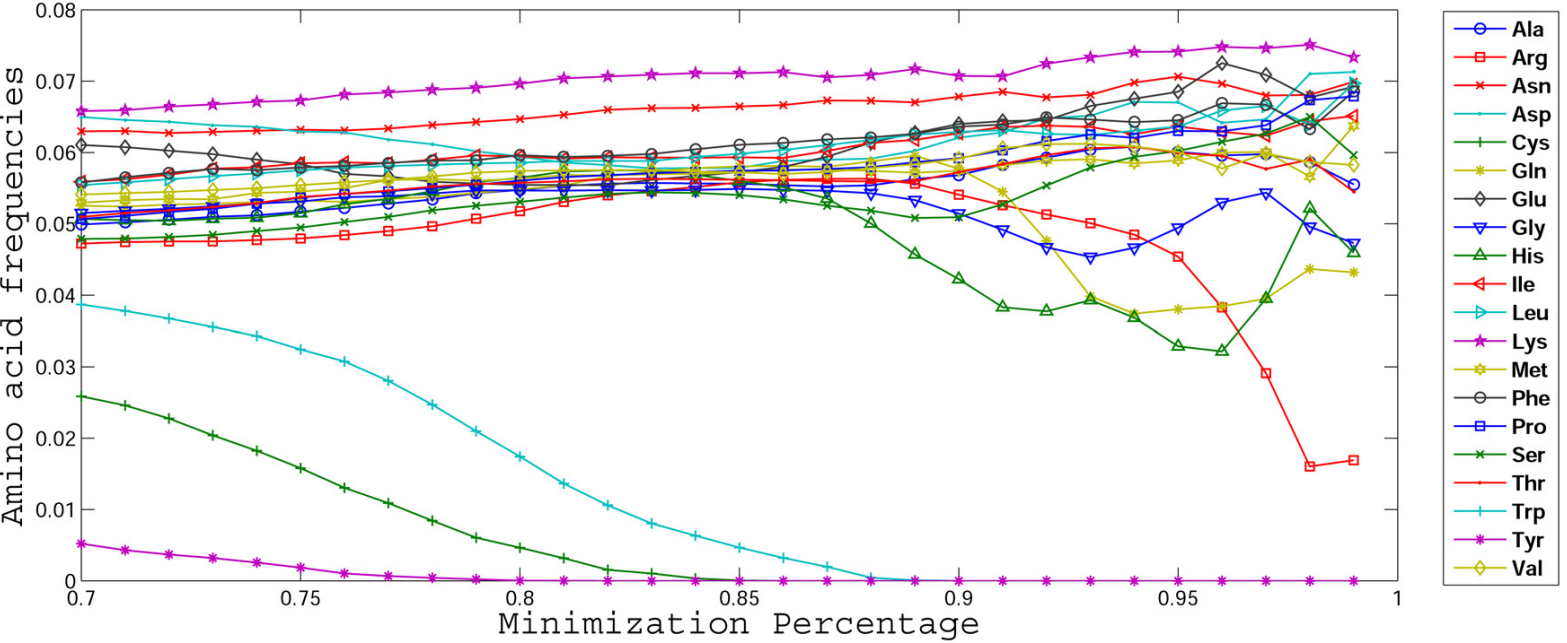
**The effects of individual amino acid assignments on the error minimization level of 2-letter codes similar to the code in Fig. 8a**. Amino acid frequencies are shown for the random codes (see text for details) for which MP is equal to or higher than the given value. For instance, there are no codes containing **Trp **which with MP > 0.9, and no codes containing **Tyr **with MP > 0.8.

Independent of the number of chosen cells *N *and the strategy that is used to fill (or not to fill) the remaining cells, the results qualitatively appear as shown in Fig. 9. The general conclusion is that the major reason of non-optimality of 2-letter codes obtained with the parsimony principle (as in Fig. 8a) are the amino acid assignments in the supercodons UAN and UAG which correspond to **Tyr**, **Cys**, and **Trp **(and two of the three stop codons) in the standard code. We were unable to discriminate the effects of other amino acids except that these effects were relatively small and sensitive to the choice of *N *(Fig. 9 and data not shown) but the non-optimality of the assignments of **Tyr**, **Cys**, and **Trp **was striking and is unambiguous (Fig. 9).

**Figure 10:**
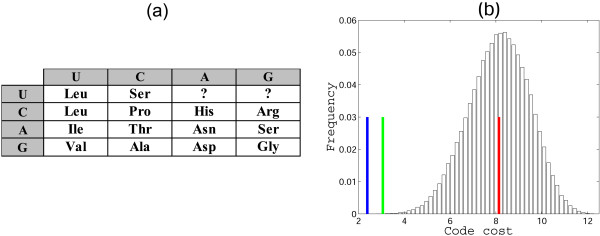
**Error minimization levels of 2-letter codes**. (a) A 2-letter genetic code obtained using the parsimony principle. For the cells with ambiguous assignment, one random amino acid is chosen; two supercodons, UAN and UGN, are disregarded; (b) The distribution of the costs of random 2-letter codes obtained by permutation of amino acid assignments in (a), the green line shows the cost of the code in (a), the red line shows the mean, and the blue line shows the optimum; MP = 0.88, the distance from the mean is 3.7 standard deviations.

Taking into account that **Tyr**, **Cys**, and **Trp **are among the 'latest' amino acids according to Trifonov's consensus of amino acid appearance [25], and that they are coded by supercodons with the lowest stability of codon-anticodon interactions (Fig. 2), it appears most likely that the primordial 2-letter genetic code did not accommodate these amino acids that were added to the amino acid repertoire only after the transition to the standard 3-letter code. Given these observations, we assessed the error minimization level of 2-letter codes without assigning the supercodons UAN and UGN (Figure 10). Such a 2-letter code is significantly more robust than the fully specified code in Figure 8a: the MP of this code is 0.88, a value that is significantly greater than the MP of the standard code (0.78), with the probability to find a better code of approximately 1/50000.

**Figure 11:**
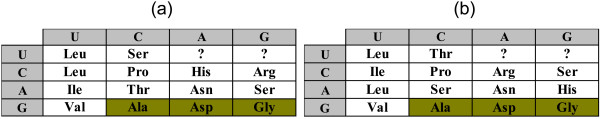
**Error minimization levels of 2-letter codes**. (a) A 2-letter code similar to that in Figure but with fixed assignments for 3 amino acids (dark green); (b) optimal code found by permutation of amino acid assignments in (a), MP = 0.91.

In the original experiment on spontaneous formation of organic compounds, Miller [14] observed detectable amounts of only three amino acids: **Ala**, **Asp **and **Gly**. In most of the subsequent abiogenic synthesis experiments, these amino acids were most abundant. Thus, it seems to be a plausible assumption that these amino acids were the first to be encoded unambiguously in the primordial code, and their positions were fixed by chance ('frozen accident' *sensu *Crick). We measured the level of error minimization for the 2-letter code, with permutations of amino acid assignments allowed only for the entries other than GCN, GAN, GGN, UAN, and UGN (Fig. 11a).

The codes in this group are not exceptionally robust to translational mistakes (MP is 0.91-0.93 depending on the choice of amino acids for the UUN, CAN, AAN, AGN supercodons). Inspection of the optimal codes readily reveals the main source of this non-optimality: in all optimal solutions **Arg **changes its position from the fourth to the third column of the table (Fig. 11b). Arginine has a prominent place in the study of the genetic code evolution. From the point of view of the adaptive theory, **Arg **is the amino acid that brings most non-optimality into the standard code [11,44,45]. At the same time, **Arg **is the amino acid for which the strongest support for a stereochemical affinity with the respective codon is available [46-49].

**Figure 12:**
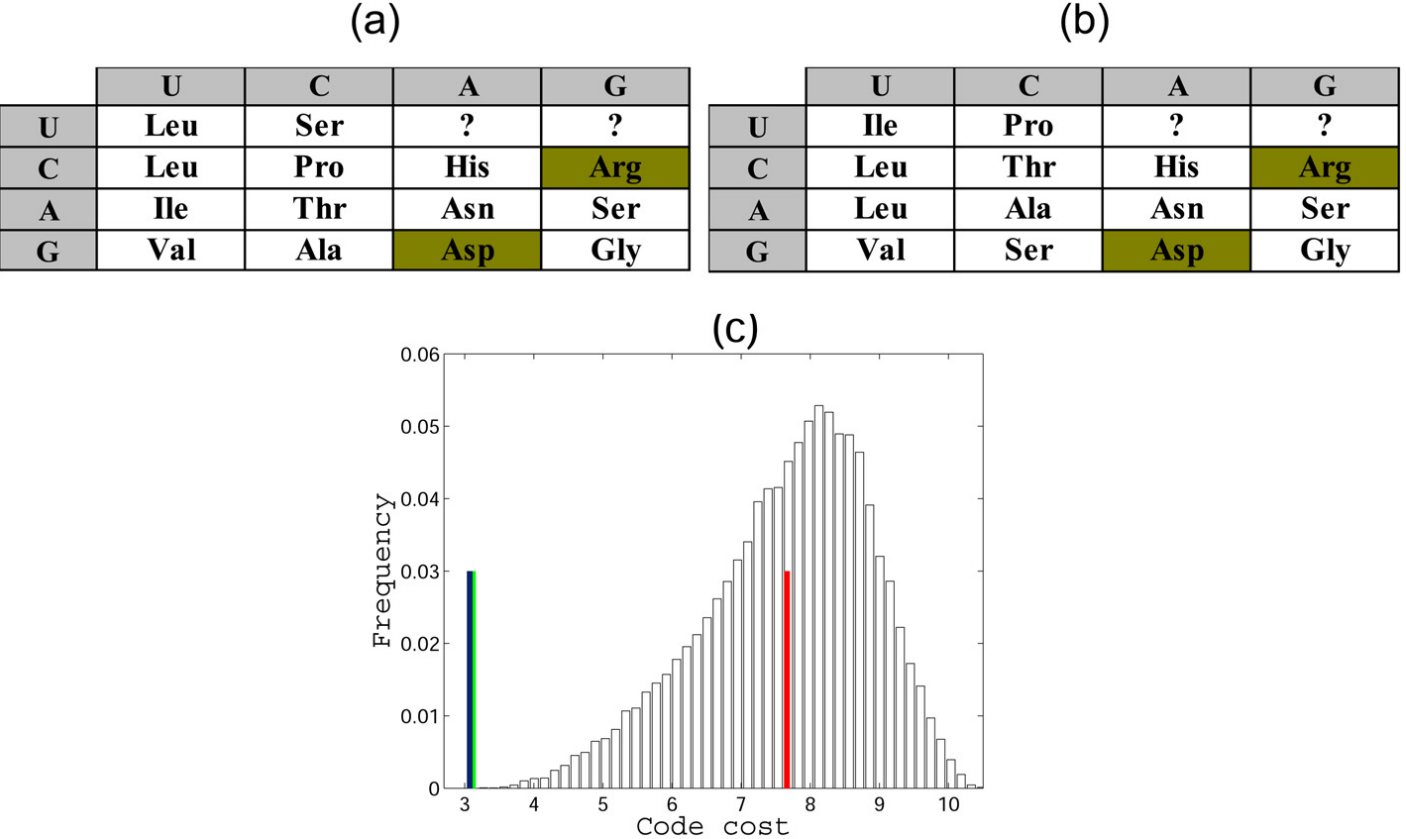
**Error minimization levels of 2-letter codes**. (a) A 2-letter code obtained using the parsimony principle, with two fixed amino acid assignments shown by dark green highlighting; (b) the result of optimization of the code by permutation of amino acid assignments in (a); (c) the distribution of the costs of random codes, the green line shows the cost of the code in (a), the red line shows the mean, and the blue line shows the optimum; MP = 0.98, the distance from the mean is 3.75 standard deviations.

Having found that the position of **Arg **is so critical for the code robustness, the following experiment was conducted. We start with the code table in Fig. 10a and the contribution of the UAN and UGN supercodons disregarded. From all other cells, two amino acids are chosen randomly and their assignments are fixed. Thus, a code table is obtained in which 4 cells are fixed (the two chosen amino acids and the supercodons UAN and UGN), whereas the assignments for the remaining 12 cells are freely permuted, and the MP is calculated for all such permutations. We found that **Arg **is unique in this setting: for most of the amino acids, pairing with **Arg **yields the highest MP of all possible pairings. The resulting MP values are all within the range of 0.89 to 0.94, with one notable exception: if the pair **Asp**-**Arg **is fixed, then MP of the code in Fig. 12a is 0.98 (the optimal code is shown in Fig. 12b).

## Conclusion

Immediately after the standard genetic code was deciphered, it has become apparent that the code table has a distinctly non-random structure, with similar codons encoding related amino acids [2,40,50,51]. An obvious and crucial question is, what are the underlying causes of this regularity?

Three major conceptual frameworks have been developed to explain the regularities in the code [8,12,52]. The error-minimization theory holds that the structure of the code is the result of selection for robustness to mistranslation [11,34,39,53,54]. The stereochemical theory posits that the code is determined, mostly, by stereochemical affinities between coding triplets (codons and/or anticodons) and the cognate amino acids [49,55,56]. The stereochemical theory alone cannot account for the high level of error minimization in the standard code; moreover, the affinity between cognate triplets and amino acids appears to be largely independent of the highly optimized amino acid assignments code [57]. To explain the structure of the code, the proponents of the stereochemical theory postulate that only part of the code is stereochemically fixed, whereas other amino acid assignments are free to be redistributed, and reassignment of even a few amino acids is sufficient for substantial optimization of the code [49]. The third, coevolution theory postulates that the structure of the code reflects the biosynthetic pathways of amino acid formation. Under this scenario, during the code's evolution, subsets of codons for precursor amino acids have been reassigned to encode product amino acids [10,21]. Then, the high level of the code optimality is just a byproduct of the evolutionary expansion in the code, and selection for robustness played only a minor role in the evolutionary shaping of the code [38] (although this role is still maintained to be more important than that of stereochemical affinities [58]). Recently, a detailed extension of the coevolution theory has been developed where the evolutionary steps of the genetic code evolution are given in details [59].

A common feature of the stereochemical theory and the coevolution theory that is central to the present study is that the level of error minimization in the primordial codes is assumed to be low but is thought to have increased to the present level as a results of late amino acid reassignments (stereochemical theory) or capture of new amino acids (coevolution theory). The results of the present analysis of 2-letter codes are at odds with this view. Under minimal additional assumptions about the primordial code, which include the lack of unique assignments for the supercodons UAN and UGN, and the assignment of **Arg **on the basis of stereochemistry, we arrive to the conclusion that the primordial 2-letter code was either shaped almost exclusively by the selective forces to minimize the impact of mistranslation or emerged in this highly robust form as a result of an extremely rare event. Indeed, with these assumptions only, combined with a random fixation of the assignment for **Asp**, we find that the 2-letter code constructed from the putative primordial amino acids using the parsimony principle is nearly optimal with respect to error minimization (MP > 0.98).

We suspect that the high robustness of the primordial code is a pre-requisite for the evolution of the translation system that was, probably, considerably more error-prone at the early stages of evolution than it is in modern organisms [7,60,61]. The subsequent expansion of the code, whether it occurred on a stereochemical basis or by coevolution led only to a decrease of the code robustness. This course of evolution was made possible by the evolution of the modern, high-fidelity translation system as well as proteins that are partially optimized for robustness to misfolding [62], and was driven by the selective advantage of the increased diversity of the amino acid repertoire.

## Methods

### The code cost

The genetic code is a mapping *a*: *C *→ *A *that assigns an amino acid (or stop signal) *a*(*c*) ∈ *A *for any codon *c *∈ *C*. The cost function can be written as

where matrix *p*(*c*'|*c*) gives the probability of misreading codon *c *as codon *c*'. The numerical values for this matrix can be obtained in different ways. We adopt the most commonly used scheme where only codon pairs that differ in one nucleotide are considered. To account for the transition-transversion bias at the levels of both mutation and translation, transitions are set to be two-fold more frequent than transversions in the first position of the codon, and five-fold more frequent in the second position. Specifically, we use the matrix *p*(*c*'|*c*):

This assumption was widely used in previous analyses of the error minimization in genetic codes (see [11,34,35] for discussion). It could be argued that recent experimental evidence shows no bias in the second base ([13,63] but more data is needed for reliable conclusions, so we adhered to the traditional scheme that was chosen a priori, before performing any simulations and numerical experiments.

The matrix *d*(*a*(*c*), *a*(*c*')) defines the cost of replacing amino acid *a*(*c*) with amino acid *a*(*c*'). The choice here is also manifold (see, e.g., [13], where a complex index is defined to estimate the cost of amino acid replacement) but we employed only one measure of amino acid similarity, namely the Polar Requirement Scale proposed by Woese et al. [64], which is a measure of amino acid hydrophobicity. The cost of replacement of one amino acid with another is calculated as *d *= (*p*(*a*) - *p*(*a*'))^2^, where *p*(*a*) and *p*(*a*') are the values of the amino acids *a *and *a*' at the Polar Requirement Scale.

Using this formalism, the cost of any genetic code can be calculated; the smaller the value, the higher the error minimization level of the given code.

### Minimization percentage of a code

To estimate the relative level of robustness to translational errors for a given code, we calculate Minimization Percentage (MP):

where *E*(*ϕ*)is the mean value of the distribution of code costs which are obtained as permutations of the amino acid assignments in the code table, *ϕ*_*code *_is the cost of the given code, and *ϕ*_*opt *_is the cost of the optimal code which can be obtained for the given set of amino acids; the criterion of optimality is robustness to translational mistakes. To find the optimal code and its cost, exhaustive search of some of the two-letter codes is possible but this search is computationally intensive for the numerous 2-letter codes that we analyzed in the course of this study. In most cases, a heuristic combinatorial algorithm was used (the Great Deluge Algorithm [65]), 3 to 5 solutions were identified, and the solution with the lowest cost was taken as the optimal.

## Competing interests

The authors declare that they have no competing interests.

## Authors' contributions

ASN and EVK conceived of the study, ASN performed the computational experiments and wrote the original draft, EVK added the biological interpretation and wrote the final version of the article that was approved by both authors.

## Reviewers' reports

Reviewer 1: Paul Higgs, McMaster University, nominated by Arcady Mushegian

Comments on 'Exceptional error minimization in putative primordial genetic codes'

At the beginning of this paper, the authors note that numerous hypotheses have been formulated regarding the genetic code but no generally accepted consensus has been reached. I am hoping that this sentence is somewhat pessimistic. On reading this paper, I find that there are a lot of important points where the authors' point of view is very similar to my own. Therefore I think it is worthwhile briefly emphasizing these things, in the hope that this consensus may spread beyond a limited few.

Firstly, this paper agrees with my own opinion (and that of many other authors) that the arrangement of the amino acids in the code is highly non-random and that some kind of selective evolutionary process is needed to generate the degree of optimality that is seen. Secondly, this paper supposes that there was a gradual addition of amino acids to the code and therefore needs to consider which amino acids were early and late additions. The early ones are taken to be the 10 that are found in Miller and Urey experiments, which have previously been assumed by authors such as Wong [10] and Di Giulio [59]. The consensus order of Trifonov [25] is also rather similar. My own recent work [20] has shown that these same amino acids turn up in other non-biological contexts, such as meteorites and other prebiotic chemistry experiments, including those intended to mimic hydrothermal vents. Thus, we can conclude that these amino acids were early without worrying about whether the details of the Miller and Urey experiments are appropriate as a description of the place and mechanism of the origin of these molecules. The early amino acids in list (1) in this paper are given precisely in the order we have proposed [20], which is related to thermodynamics. The simplest amino acids (like Gly and Ala) are thermodynamically least costly to form, and occur in much greater quantities in non-biological contexts than do the amino acids at the end of the early list (Pro, Thr). Thirdly, there seems to be a consensus that the build up of the code occurred over the same time period that the biochemical pathways for amino acid synthesis were evolving. It is therefore likely that amino acids that are formed at the end of long synthetic pathways are late additions to the code. This point is central to the coevolution theory [10,59,66], and is also consistent with the view in this paper and in my own work [13], even though I have argued against the details of the way that the precursor-product relationships are used to infer the ancestral code in the coevolution theory. Fourthly, this paper supposes that the code was always a triplet code, but that the three letters may not all have encoded information initially. This paper supposes the first two letters were important and the third was redundant. I have proposed an earlier stage in which only the second letter was important and the code had a four-column structure [13]. However, it seems entirely plausible that a two-letter code could evolve from the four-column code, or at least that there was an intermediate stage with around 10 amino acids where almost all amino acids were encoded by four-codon families and not two-codon families. Thus, it seems to me that this level of consensus on all the above points provides some grounds for optimism.

Authors' response: *We appreciate these constructive comments and agree that the emerging consilience of different approaches to the code evolution problem is highly encouraging. An excellent case in point is the order of amino acid appropriation for protein synthesis emphasized by Higgs: indeed, we do not have to worry too much about whether or not the conditions of the Miller-Urey experiments were realistic with respect to the primordial earth environment or not (it is essentially certain that they were not) because other experiments and estimates yield virtually indistinguishable results. Indeed, this consensus makes us be cautiously optimistic about the possibility of attaining some certainty in this difficult area of research and encourages us to continue with our investigation of code evolution*.

The main disappointment I have with this paper is that it makes use only of the polar requirement scale to calculate the cost of amino acid replacement. Even though polar requirement seems to be an important property, I do not believe that any one property is sufficient. Furthermore, there are some particular problems with polar requirement, in that Gly, Arg, and Trp turn out to fortuitously have rather similar polar requirements, whereas these amino acids are very different from one another according to almost all other properties. This makes a substantial difference to the degree of optimality. The amino acid distance function I have derived in [13] weighs the effects of several physical properties in a principled way, and is derived from maximum likelihood fitting of amino acid substitution data. I really feel this is much better than the polar requirement scale. Polar requirement was sufficient to demonstrate that the code was optimized to some degree, but now we have moved beyond that. If we are interested in the details, as in this paper, then a better cost function is required.

**Author's response**: *We do not know what "really" defines the similarity between amino acids or the cost of amino acid replacements when the genetic code is analyzed. The Polar Requirement scale is the measure that seems to express this similarity remarkably well in the context of code optimization. Indeed, it has been shown that there is no better amino acid similarity measure in terms of revealing the optimality of the standard genetic code when compared with random alternatives *[26]. *This very observation, in our opinion, allows us to use this scale for the cost function without necessarily seeking a 'better' amino acid similarity measure. With regard to the specific example considered by Higgs, it is difficult to agree that Gly, Arg and Trp have similar polar requirements. Indeed*, ***Trp****has the value 5.2 on the Polar Requirement scale*, ***Gly****has a value of 7.9, and ****Arg****has a value of 9.1. Thus, these amino acids span approximately 50% of the total range of Polar Requirement values (from 4.8 for ****Cys****to 13 for ****Asp***). *As the side chain of ****Arg****is rather hydrophobic, it would seem inappropriate for the amino acid to be positioned at the polar end of the scale. Thus, the difference in the Polar Requirement values seems to reasonably reflect the difference between the properties of these amino acids. Having said all this, we cannot rule out that the scale developed by Higgs *[13]*is indeed a better metric for comparing amino acids than the Polar Requirement alone. However, the analysis described in this article was initiated and partially completed prior to the publication of Higgs' work, and at this point, redoing it completely using a different scale is impractical. We hope to compare the results obtained with the two scales in a future study*.

An interesting aspect of this paper is Figure 2, which shows that the early amino acids are assigned to the codon boxes with the strongest interactions at the first two codon positions. The thermodynamic information used here is taken from Travers [30], who takes it from Protozanova et al. [67]. However, these measurements come from stacking of DNA not RNA, *i.e*. the backbone is different and T bases are present, not U. A thermodynamic model for staking of RNA base pairs is available and is commonly used in RNA structure prediction [68]. The RNA measurements are derived from melting of oligomer duplexes. They are not specific to the geometry of the codon-anticodon interaction, and are not ideal for the present purposes. However, at least they are measured with RNA. I do not understand why Travers [30] and the present authors should chose to base their conclusions on the DNA parameters rather than the standard RNA ones.

In Table 1, amino acids are listed in the order given by the DNA melting data of [30], which is the same order as Figure 2. However, the ranking from 1 to16 in Figure 2 obscures the point that several of the pairs are related by symmetry. For example GU is complementary to AC, and therefore the figures for Val and Thr must be the same. Similarly CU (Leu) must be the same as AG (Ser/Arg). So it is not really true that Arg is the sole exception. We should also ask why the AG box does not contain an early amino acid with a four-codon family, and why the AU box contains both Ile and Met.

**Table 1:** DNA and RNA stacking energies for the first 2 codond letters and the strong-weak codon classification for the 20 amino acids

1st/2nd base	Amino acid	**DNA melting **[30,67]	**RNA stacking **[68]	**S/W theory **[69]
GC	Ala	-2.70	-3.42	SSS
GU	Val	-2.04	-2.24	SWS
AC	Thr	-2.04	-2.24	WSS
GG	Gly	-1.97	-3.26	SSW
CC	Pro	-1.97	-3.26	SSS
GA	Asp/Glu	-1.66	-2.35	SWW
UC	Ser	-1.66	-2.35	WSS
CG	Arg	-1.44	-2.36	SSW
CU	Leu	-1.29	-2.08	SWS
AG	Ser/Arg	-1.29	-2.08	WSW
AU	Ile/Met	-1.27	-1.10	WWS
UU	Phe/Leu	-1.04	-0.93	WWS
AA	Asn/Lys	-1.04	-0.93	WWW
CA	His/Gln	-0.78	-2.11	SWW
UG	Cys/Trp	-0.78	-2.11	WSW
UA	Tyr/Stop	-0.12	-1.33	WWW

The RNA stacking figures from Xia et al. [68] are also given (Table 1). The order differs from the order in Figure 2 to a considerable extent, although the bottom line is the same. There is a tendency for the early amino acids to be assigned to four-codon families where the first two bases have strong stacking interaction. This general point seems to be true for both sets of parameters, but the correlation is not perfect in either case. I think that we cannot say anything more concrete than this unless thermodynamic parameters that are more specific to the geometry of the anticodon loop become available.

I have been thinking of these RNA stacking parameters recently in the context of translational selection. Our work in progress shows that weak interactions tend to correspond to cases of strong codon bias, presumably because weak interactions are associated with slow recognition of the incoming tRNA by the ribosome; hence there is slow translation and a large potential for selection to act between synonymous codons. Among the two-codon U+C amino acids, Phe and Asn appear to have the strongest codon bias, and these have the weakest interaction according to the RNA parameters, whereas according to the DNA parameters, Tyr, Cys and His would be weaker than this. Thus it seems that our observations on codon bias can be explained better with the RNA parameters than the DNA ones, which is perhaps not surprising as the molecules involved are RNAs!

A further twist that deserves to be mentioned is the argument of Lehmann and Libchaber [69] regarding the stability of the anticodon loop. For each anticodon-codon interaction, they consider three factors, each of which is classified as strong (S) or weak (W). Factors 1 and 2 correspond to the base pairs occurring at the first and second codon positions. These are always Watson-Crick pairs. GC pairs are counted as S and AU pairs as W. The third factor is the additional stability of the anticodon loop that occurs if the middle base (position 35 in the tRNA alignment) is a purine. At purine at position 35 is able to hydrogen bond with a conserved U base at position 33 (one base upstream of the anticodon). Thus tRNAs with purines at position 35 are classed as S and those with pyrimidines are classed as W. The S/W classification for each of the four-codon boxes is given in Table 1.

It can be seen that the cases where there are two or three S factors correspond to four-codon families whereas those where there are zero or one S correspond to cases where the codon box is split between two amino acids. Lehmann and Libchaber [69] suggest that splitting up a four-codon family is not possible if the anticodon-codon interaction is strong because a single tRNA would be able to interact with all four codons in this case. In fact, in bacterial and mitochondrial genomes, this is known to occur when the wobble base of the tRNA is U. In contrast, if the interaction is weaker at the first two codon positions then a more specific interaction at the third position may be required. Hence at least two tRNAs with different wobble position bases are required (usually a U and a G), and there is the possibility of splitting the codon box between two different amino acids. From the table above, it seems that Lehmann and Libchaber's S/W classification explains the distribution of the amino acids at least as well as the two free energy scales.

It is worth emphasizing the connection between the different points made above. The early amino acids may have been assigned to strongly interacting codons because these were the easiest ones to use for an early translation system that was still poorly adapted and error-prone. The more weakly interacting codons may have been incorporated later when the translation system evolved to become more efficient and accurate; hence the later amino acids are assigned to the weakly interacting codons. However, in the later stages of genetic code evolution, the ability of tRNAs with strong interactions to pair with all four codons in a family prevented the subdivision of these families into two. Hence early amino acids remain assigned to four codon families. The only exception to this is Asp/Glu.

**Author's response**: *We appreciate these considerations and have no doubts that they will be helpful to a reader interested in code evolution and the physical and selective factors that underlie it. Again, we find comments particularly helpful in view of future work where we expect to make an effort to account for stacking parameters as accurately as possible*.

Reviewer 2: Rob Knight, University of Colorado

In this manuscript, the authors investigate the hypothesis that the genetic code was optimized at an earlier stage of evolution in which only the first two codon positions were used for coding. They construct a simplified genetic code table in which the third position nucleotide is always fully degenerate, using several sources of evidence as a guide. Using a cost function that they have used for previous analyses, they show that this simplified code is much better at minimizing the effects of errors than are permuted versions of the same code. The results are interesting and provide additional evidence that the genetic code was likely optimized at an earlier stage of evolution than the present (e.g. Marquez et al. 2005 and other work that shows that current genetic messages are not optimized), and for the hypothesis that the code evolved from a simpler form in which the third codon position was not read.

The list of early amino acids is plausible and derived from a combination of the Murchison meteorite, prebiotic synthesis and metabolic pathway evidence, which are the best available data and show remarkable consensus. The parsimony rules also seem reasonable and well-specified. The observation that the putatively early amino acids seem to be most highly optimized is intriguing, and, as the authors note, most previous attempts to reconcile adaptation in the code with other mechanisms have assumed that adaptation was a relatively late overlay in a code where the earliest amino acids were fixed by other principles. The suggestion that stereochemistry played a greater role in the assignment of later amino acids is interesting, and to my knowledge novel. It will be fascinating to see whether additional amino acids for which strong stereochemical evidence is found tend to be on the "early" or "late" list. It would be a useful addition to the manuscript if the authors could explain how late stereochemical entry of amino acids could be reconciled with Wolf & Koonin's 2007 model of code evolution. The idea that the code's robustness peaked at an early stage and has since been declining is also interesting and may provide a fruitful inspiration for future modeling efforts.

One minor quibble: the manuscript states that the stereochemical theory and the coevolution theory "cannot account for the high level of error minimization in the standard code". This isn't quite right: if either the stereochemical theory or the coevolution theory were to fully explain the code table (although only the most ardent proponents of either theory would claim that this is the case, as most researchers in the field now take a pluralist approach) then the high level of error minimization would be explained as a side-effect of the real underlying mechanism and not as something that required an adaptive explanation in itself (in the same way that the red color of blood is a side-effect of selection for its ability to carry oxygen, not an adaptation in and of itself).

**Author's response**: *We agree that the code's robustness, in principle, could emerge as a non-adaptive by-product of stereochemistry or co-evolution. What is meant in the text is that, the available evidence in support of, say, the stereochemical theory, it appears insufficient to account for the error minimization properties of the standard code*.

Overall, this is an interesting and well-executed piece of research that provides new insight into the tangled pathways by which the genetic code apparently evolved.

Reviewer 3: Sandor Pongor, ICGEB

Novozhilov and Koonin present a simple mathematical model to investigate the error-minimization properties of putative primordial codes consisting of 16 supercodons. They show that the putative, primordial two-letter genetic codons encoding a 10 amino acid repertoire were nearly optimal in terms of error minimization, and conclude that that this robustness could be the outcome of extensive early selection during the co-evolution of the code with the primordial, error-prone translation system. Under this hypothesis, the subsequent expansion 3 letters resulted in a more adaptable (less error-minimized) genetic code that became sustainable owing to the evolution of a high-fidelity translation system. This is an interesting and important piece of work that also points to the origins of tunable evolvability which is, in my mind, a fascinating property of modern organisms.
